# Supplementary material for: Appropriate relaxation of non-pharmaceutical interventions minimizes the risk of a resurgence in SARS-CoV-2 infections in spite of the Delta variant
Source: PLoS Comput Biol. 2022 May 16;18(5):e1010054. doi: 10.1371/journal.pcbi.1010054 (PMC9135349; doi:10.1371/journal.pcbi.1010054)
Supplement: S1 Appendix — (PDF) [file pcbi.1010054.s001.pdf]

Table 1: NPI parameters for the different scenarios and time spans.

Parameters for baseline NPI before opening.		
	Contact reductions ($l = 1$)	Protection effects ($l = 2$)
home	0	0
school	0	[0.25,0.35]
work	0	[0.25,0.35]
other	0	[0.25,0.35]
Parameters for dynamic NPIs if threshold 35 is exceeded locally.		
	Contact reductions ($l = 1$)	Protection effects ($l = 2$)
home	[0.15,0.25]	0
school	[0.25,0.35]	[0.25,0.35]
work	[0.15,0.25]	[0.25,0.35]
other	[0.25,0.35]	[0.25,0.35]
Parameters for dynamic NPIs if threshold 100 is exceeded locally.		
	Contact reductions ($l = 1$)	Protection effects ($l = 2$)
home	[0.55,0.65]	[0.05,0.15]
school	[0.45,0.55]	[0.25,0.35]
work	[0.25,0.35]	[0.25,0.35]
other	[0.75,0.85]	[0.25,0.35]
Parameters for baseline NPI after opening.		
	Contact reductions ($l = 1$)	Protection effects ($l = 2$)
home	0	0
school	0	0 (S1, S3)<br>[0.25,0.35] (S2, S4)
work	0	0 (S1, S3)<br>[0.25,0.35] (S2, S4)
other	0	0 (S1, S3)<br>[0.25,0.35] (S2, S4)
Parameters for common NPIs in future scenarios.		
	Contact reductions ($l = 1$)	Protection effects ($l = 2$)
home	0	0
school	[0.05,0.15] (S1F)<br>[0.35,0.45] (S2F, S3F)	[0.25,0.35]
work	[0.05,0.15] (S1F, S2F)<br>[0.35,0.45] (S3F)	[0.25,0.35]
other	[0.05,0.15] (S1F)<br>[0.35,0.45] (S2F, S3F)	[0.25,0.35]
