# Supplementary material for: Appropriate relaxation of non-pharmaceutical interventions minimizes the risk of a resurgence in SARS-CoV-2 infections in spite of the Delta variant
Source: PLoS Comput Biol. 2022 May 16;18(5):e1010054. doi: 10.1371/journal.pcbi.1010054 (PMC9135349; doi:10.1371/journal.pcbi.1010054)
Supplement: S2 Appendix — (PDF) [file pcbi.1010054.s002.pdf]

Initialization of compartment models from confirmed cases, deaths, ICU and vaccination numbers

In order to run our compartment models, start values for compartments such as exposed, carrier (asymptomatic and presymptomatic), or hospitalized who are not detected or not reported have to be extrapolated from reporting of confirmed cases. For the sake of simplicity, we consider a generic age group and generic region and, thus, drop any corresponding indices. The following applies to any age group or region by taking the age- and region-specific reported numbers and parameter values.

Let us denote $\Sigma^C(t)$ the accumulated, total number of confirmed cases up to day t. Further denote $\Sigma^D(t)$ the accumulated, total number of reported deaths and $\Sigma^V(t)$ the accumulated, total number of full vaccinations up to day t. Finally, denote $\sigma^U(t)$ the number of individuals in intensive care units at day t. These values can be obtained from the official sources [1–3]. For the sake of simplicity, we assume a detection ratio of one in the following explanations. For a detection ratio $d < 1$, the corresponding factor $1/d$ has to be applied to the Σ-terms in the subsequent equations. Likewise, we can in principle apply time-dependent detection ratios $d(t)$.

Since the German national test strategy was in large parts of the pandemic essentially or primarily based on testing symptomatic individuals [4], we simply assume the number of confirmed cases to be equal to the number of symptomatic cases. In the Monte Carlo runs, we can implement an incidence-dependent detection ratio also for carriers. For more details; see [5, 6].

The number of confirmed cases represents individuals who are either currently infected or who had been infected previously. We need to consider the confirmed cases of the future with respect to the starting date of the simulation in order to determine the number of exposed and carrier individuals. Note that this is not an issue for the algorithm since we always simulate from some day in the recent past to validate the fit of, e.g., symptomatic individuals at the beginning of the simulation. Let us denote t_0 the start day of the simulation.

Models without vaccination and reinfection

We have to distinguish three cases. First, let us distinguish compartments that correspond to infection states before detection (e.g., exposed or carrier) and compartments that can be detected or happen after detection (e.g., infected or hospitalized). Compartments before detection are then again divided between individuals that will get infected (and detected) and those that recover before possible detection (e.g., the course of the "disease" *exposed* $\rightarrow$ *carrier* $\rightarrow$ *recovered*).

As in the paper, let $\mu_{*_1}^{*_2}$ denote the probability of transition from compartment $*_1$ to $*_2$ and $T_{*_1}^{*_2}$ the time span in days an individual spends in compartment $*_1$ before going to $*_2$. Note that since we use mean-value approaches, we only consider the average time between two infection states.

We use the term *presymptomatic* for carrier individuals who will develop symptoms and *asymptomatic* for carrier individuals that will directly recover with turning symptomatic.

Let us first visualize the case of carrier individuals that will become symptomatic. Individuals who fall into this carrier category at t_0 have turned carrier between t_0 and $t_0 - T_C^I$. Thus, they will turn symptomatic at the latest at $t_0 + T_C^I$; cf. the shaded area in Fig. 1. To better understand this, let us consider the edge cases of individuals who turned carrier at either $t_0 - T_C^I$ or t_0. In these cases, exposure happened at

$$\begin{aligned} t_{E,1} &:= t_0 - T_C^I - T_E^C + \varepsilon, \\ t_{E,2} &:= t_0 - T_E^C + \varepsilon, \end{aligned} \tag{1}$$

where $0 < |\varepsilon| \ll 1$. We visualized four different courses of the disease with moment of exposure at $t_{E,1}$ and $t_{E,2}$ for $\varepsilon > 0$ and $\varepsilon < 0$; see Fig. 1. Blue and gold individuals are carrier at t_0 while the red individual is already symptomatic before t_0 and and the purple one only turns carrier after t_0. Hence, the number of confirmed cases $\Sigma^C(t_0 + T_C^I) - \Sigma^C(t_0)$ are carrier individuals at t_0.

For the exposed, we consider the moments of exposure

$$\begin{aligned} t_{E,2} &= t_0 - T_E^C + \varepsilon, \\ t_{E,3} &:= t_0 + \varepsilon. \end{aligned} \tag{2}$$

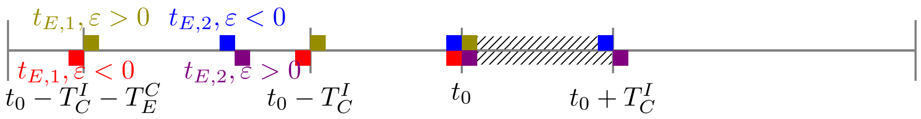

Figure 1: Presymptompatic individuals at t_0 will develop symptoms between t_0 and $t_0 + T_I^C$ and can then be confirmed by testing symptomatic individuals in the shaded area. Visualization of four courses of the disease (CODs; red, olive, blue, and purple) with moment of exposure $t_{E,1}$ and $t_{E,2}$. The first rectangle in each color represents exposure, the second the moment turning carrier and the third the moment turning symptomatic (infected). For visualization, we use $T_E^C = 3$ and $T_C^I = 2$.

cf. Fig. 2 shows four different CODs. Now, individuals purple and red are exposed at t_0 and can be confirmed between $t_0 + T_C^I$ and $t_0 + T_E^C + T_C^I$. The blue individual is already carrier at t_0 while the golden only gets exposed after t_0. Thus, $\Sigma^C(t_0 + T_E^C + T_C^I) - \Sigma^C(t_0 + T_C^I)$ are part of the exposed population at t_0.

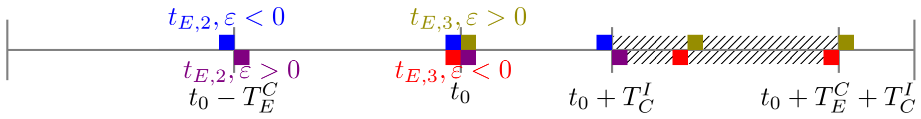

Figure 2: Exposed individuals at t_0 who will develop symptoms will do so between $t_0 + T_C^I$ and $t_0 + T_E^C + T_I^C$. Similar to Fig. 1, we visualize four CODs.

Secondly, we have carrier individuals who will directly recover from an asymptomatic infection and never develop symptoms. We now estimate the initial number of carrier and exposed individuals that will never turn symptomatic. Note that the testing strategy defines if these can be confirmed or not at any point of the asymptomatic infection. As we laid out before, we assume that asymptomatic cases are, in majority, not tested.

The number of asymptomatic individuals at t_0 that will eventually recover without symptoms depends on the time span T_C^R and on the share of asymptomatic infections among all infections μ_C^R. The number of asymptomatic individuals recovering at t is proportional to the number of eventually symptomatic individuals that were exposed at the same day as the asymptomatic cases.

In order to find the interval of exposure of asymptomatic carrier individuals at t_0, we use two moments of exposure

$$\begin{aligned} t_{E,2} &:= t_0 - T_E^C + \varepsilon, \\ t_{E,4} &:= t_0 - T_C^R - T_E^C + \varepsilon, \end{aligned} \tag{3}$$

where $0 < |\varepsilon| \ll 1$. The asymptomatic individual that was exposed at $t_{E,4}$ will recover at

$$t_{E,4} + T_E^C + T_C^R = t_0 + \varepsilon. \tag{4}$$

The asymptomatic individual that was exposed at $t_{E,2}$ will turn asymptomatic at

$$t_{E,2} + T_E^C = t_0 + \varepsilon. \tag{5}$$

Hence, for $\varepsilon < 0$, the individual exposed at $t_{E,4}$ is already recovered before t_0 (and not anymore carrier). On the other hand, for $\varepsilon > 0$, the individual exposed at $t_{E,2}$ is still in exposed and not yet carrier state at t_0. We conclude that carrier individuals at t_0 which recover without symptoms have been exposed between $t_0 - T_C^R - T_E^C$ and $t_0 - T_E^C$. As before, we visualize four CODs in Fig. 3.

We now estimate the number of asymptomatically recovering individuals from their counterparts symptomatic counterparts who were exposed in the same range. The presymptomatic individual that was exposed between $t_0 - T_C^R - T_E^C$ and $t_0 - T_E^C$ will turn symptomatic between

$$t_0 + T_C^I - T_C^R \quad \text{and} \quad t_0 + T_C^I;$$

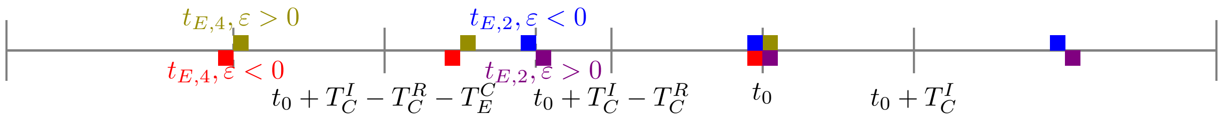

Figure 3: Four asymptomatic courses of the disease (red, olive, blue, and purple), i.e., individuals that will not turn symptomatic. The three rectangles now represent exposure, turn to carrier and recovery (instead of turning symptomatic). For visualization, we use $T_E^C = 3$, $T_C^I = 2$ and $T_C^R = 4$.

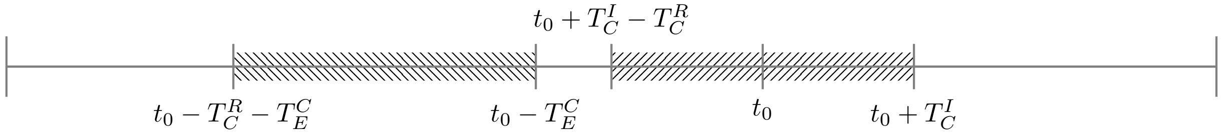

Figure 4: Individuals who got exposed in the left shaded area and who eventually will develop symptoms and will turn symptomatic in the right shaded area.

cf. Fig. 4; from now on, we skip visualization of different CODs. We then approximate the number of asymptomatic carriers (i.e., who will not develop symptoms) from their coinfected presymptomatic counterparts (i.e., who will develop symptoms). Thus, we add

$$\frac{\mu_C^R}{1-\mu_C^R}(\Sigma^C(t_0 + T_C^I) - \Sigma^C(t_0 + T_C^I - T_C^R))$$

to the previously obtained number of presymptomatic carriers.

Exposed individuals at t_0 that will directly recover from asymptomatic infections have to be estimated from exposed individuals at t_0 that will eventually develop symptoms. Since T_E^C is the common time of both groups to turn carrier after exposure, we add

$$\frac{\mu_C^R}{1-\mu_C^R}(\Sigma^C(t_0 + T_E^C + T_C^I) - \Sigma^C(t_0 + T_C^I))$$

to the previously obtained number of exposed individuals; cf Fig. 2.

We then set initial values for exposed and carriers as

$$E(t_0) = \frac{1}{1-\mu_C^R}(\Sigma^C(t_0 + T_E^C + T_C^I) - \Sigma^C(t_0 + T_C^I)), \tag{6}$$

$$C(t_0) = \frac{\mu_C^R}{1-\mu_C^R}(\Sigma^C(t_0 + T_C^I) - \Sigma^C(t_0 + T_C^I - T_C^R)) + (\Sigma^C(t_0 + T_C^I) - \Sigma^C(t_0)). \tag{7}$$

To summarize, the first part of equation (7) describes individuals that will have an asymptomatic infection and not turn symptomatic. The second part describes the number of individuals that will develop a symptomatic infection.

The third case in initialization corresponds to compartments at or after (possible) detection. The equation for the infected compartment (i.e., symptomatic) is obtained easily. With Fig. 5 and Fig. 6, we obtain the initialization:

$$I(t_0) = \mu_I^H(\Sigma^C(t_0) - \Sigma^C(t_0 - T_I^H)) + (1-\mu_I^H)(\Sigma^C(t_0) - \Sigma^C(t_0 - T_I^R)). \tag{8}$$

The infected compartment is obtained from individuals that become hospitalized later on $((\mu_I^H))$ and those who only experience a mild infection $((1-\mu_I^H))$.

For hospitalized patients at t_0, we add again an additional layer and obtain

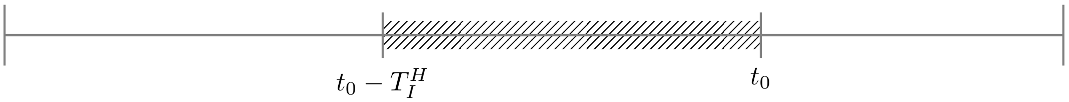

Figure 5: Symptomatic individuals at t_0 that will need hospitalization develop symptoms at the earliest at $t_0 - T_I^H$. Otherwise, they are already hospitalized at t_0.

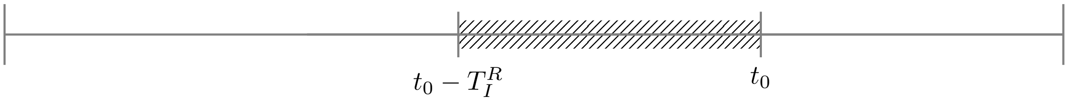

Figure 6: Symptomatic individuals at t_0 that will not need hospitalization (i.e., recover from a mild symptomatic COD) develop symptoms at the earliest at $t_0 - T_I^R$. Otherwise, they are already recovered at t_0.

$$H(t_0) = \mu_I^H(\mu_H^U(\Sigma^C(t_0 - T_I^H) - \Sigma^C(t_0 - T_I^H - T_H^U)) + (1 - \mu_H^U)(\Sigma^C(t_0 - T_I^H) - \Sigma^C(t_0 - T_I^H - T_H^R))); \tag{9}$$

see also Fig. 7 and Fig. 8.

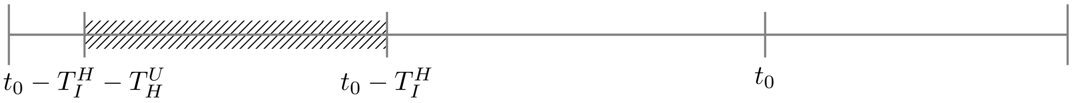

Figure 7: Hospitalized individuals at t_0 that will need ICU treatment develop symptoms at the earliest at $t_0 - T_I^H - T_H^U$ and at the latest at $t_0 - T_I^H$.

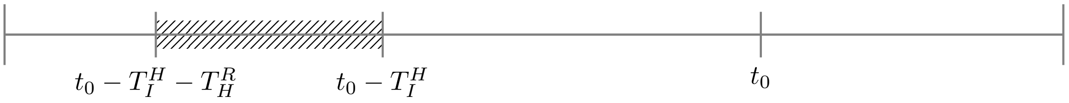

Figure 8: Hospitalized individuals at t_0 that will not need ICU develop symptoms at the earliest at $t_0 - T_I^H - T_I^R$ and at the latest at $t_0 - T_I^H$.

In theory, the number of ICU patients could be obtained accordingly if no data was available for this. Here, we take the daily report by [3] and set

$$U(t_0) = \sigma^U(t_0). \tag{10}$$

Deaths are reported as well. However, note that in [1], the reported date does not correspond to the day of the death but to the day the infection of the deceased was assumed, so a time shift of numbers is needed. We consequently set

$$D(t_0) = \Sigma^D(t_0 - T_I^H - T_H^U - T_U^D). \tag{11}$$

Individuals that have already recovered from the disease can be computed by taking the confirmed cases of the simulation start date and subtract all other symptomatic individuals at t_0 that have not (yet) recovered:

$$R(t_0) = \Sigma^C(t_0) - I(t_0) - H(t_0) - U(t_0) - D(t_0). \tag{12}$$

Finally, the number of susceptible is set as

$$S(t_0) = P - E(t_0) - C(t_0) - I(t_0) - H(t_0) - U(t_0) - R(t_0) - D(t_0), \tag{13}$$

where P is the population of the specific region and age group.

Models with vaccination or reinfection

In case of vaccination or possible reinfection, reported case numbers have to distributed accordingly to the subpopulations (e.g., vaccinated and unvaccinated) at the beginning of the simulation. Let us assume that we have $k+1$ subpopulations denoted by index $Y_0, \dots, Y_k$ which we distinguish by a different level of immunization, e.g., each subpopulation may have a different number of vaccine doses and recoveries from (multiple) infections. For simplicity, Y_0 will be the yet unvaccinated and uninfected subpopulation.

In our paper [6], we use Y_0 as well as $Y_1 = PV$ (one dose of vaccination) and $Y_2 = V$ (two doses of vaccination, recovery from previous infection(s), or a combination of these).

To introduce subpopulation specific parameters, we add the subindex Y_m to the compartments names E, C, I, H, and U of the previous section. For instance, $E_{Y_0}(t_0)$ will be the exposed individuals at t_0 that have not been vaccinated nor had any infection before and $T_{E_{Y_0}}^{C_{Y_0}}$ will be the (average) time the individual stays exposed before turning to carrier state.

Denote by $N_{Y_m}^{D^\perp}$ the sum of all living individuals in subpopulation Y_m, $m = 0, \dots, k$. In our case, we obtain the number of partially and fully vaccinated individuals from [2]. The share of subpopulation m in the total population at t_0 then is

$$\frac{N_{Y_m}^{D^\perp}(t_0)}{\sum_{m=0}^{k} N_{Y_m}^{D^\perp}(t_0)}. \tag{14}$$

In an optimal world, we would continue with (14). This means that we need a continuous reporting of deaths D_{Y_m} per subpopulation. This, however, is not given for the German counties and the different vaccination or recovery states as used in [6]. Since $N_{Y_m}^{D^\perp}$ is unknown for considered regions and since the number of SARS-CoV-2 related deaths compared to the total population size is small, we consider

$$\frac{N_{Y_m}(t_0)}{\sum_{m=0}^{k} N_{Y_m}(t_0)}, \tag{15}$$

where $N_{Y_m} \approx N_{Y_m}^{D^\perp}$ is the sum of all individuals in subpopulation m and that may have died in the recent past. For $Y_1 = PV$, we have

$$N_{Y_1}(t_0) = \Sigma^V(t_0 - 14 + T_{PV}^V), \tag{16}$$

where T_{PV}^V is the averaged time span between first and second dose of the vaccine, $\Sigma^V(t)$ the reported number of full vaccinations up to day t, and 14 the number of days for the first vaccination to take full effect; cf [6]. However, for $m \in \{0, 2\}$, $N_{Y_m}(t_0)$ is not directly available in our case. In our model [6], we make the simplifying assumption that multiple recoveries from infections equates to recovery from a single infection and that recovery equates to full vaccination. This means that we have to estimate the number of recovered individuals to obtain $N_{Y_2}(t_0)$. Of course, we have

$$P = N_{Y_0}(t) + N_{Y_1}(t) + N_{Y_2}(t), \quad \forall t, \tag{17}$$

where P is the population of the specific region and age group. Consequently, we have to find either $N_{Y_0}(t_0)$ or $N_{Y_2}(t_0)$ to use (15). Since the day of recovery is not reported in [1], we do not have the recovered individuals $R(t)$ up to day t for any region. In order to initialize the population size, we make the rough approximation

$$N_{Y_2}(t_0) = \Sigma^C(t - 14) + \Sigma^V(t - 7), \tag{18}$$

where $\Sigma^C(t-14)$ is based on the assumed recovery time span of 14 days for mild infections [1] (which make the major part of the overall infections). $\Sigma^V(t-7)$ means that we take a time span of seven days for the full vaccination to take effect; cf. [6].

Note that $\Sigma^V(t-14) + \Sigma^C(t-7)$ may count individuals twice that get reinfected or infected after full vaccination. In order to account for this, correspondingly resolved data is needed or approximations have

to be done to obtain a better initialization than (28). The more that waning immunity or the Omikron variant are considered, this can become a real issue. However, for the model scenarios in [6], this was not yet considered.

Equation (15) is not yet a good approximation for the share of daily new infections since each group has different protective factors against asymptomatic, symptomatic, or severe infections. For $m = 0, \dots, k$ and compartment X_{Y_m}, respectively, we introduce the factors $p_{X_{Y_m}}$ as

$$P(X_{Y_m}) = p_{X_{Y_m}} P(X_{Y_0}). \tag{19}$$

Here, $P(X_{Y_m})$ represents the probability of an individual of subpopulation Y_m to undergo state X_{Y_m}. We have $p_{X_{Y_0}} = 1$ for all disease states. For more details, see equation (30) in [6].

The number of new infections at day t and in subpopulation m is then approximated by

$$\frac{N_{Y_m}(t)\, p_{I_{Y_m}}}{\sum_{m=0}^{k} N_{Y_m}(t)\, p_{I_{Y_m}}} (\Sigma^C(t+1) - \Sigma^C(t)). \tag{20}$$

Based on (6)–(10) and the weighted subpopulation reduction factors for different states of the disease, we approximate the initial values for subpopulation m by

$$E_{Y_m}(t_0) = \frac{N_{Y_m}(t_0)\, p_{E_{Y_m}}}{\sum_{m=0}^{k} N_{Y_m}(t_0)\, p_{E_{Y_m}}} \left[\frac{1}{1-\mu_{C_{Y_m}}^{R_{Y_m}}} \Big(\Sigma^C(t_0 + T_{E_{Y_m}}^{C_{Y_m}} + T_{C_{Y_m}}^{I_{Y_m}}) - \Sigma^C(t_0 + T_{C_{Y_m}}^{I_{Y_m}}) \Big) \right], \tag{21}$$

$$\begin{aligned} C_{Y_m}(t_0) = {} & \frac{N_{Y_m}(t_0)\, p_{C_{Y_m}}}{\sum_{m=0}^{k} N_{Y_m}(t_0)\, p_{C_{Y_m}}} \left[\frac{\mu_C^R}{1-\mu_{C_{Y_m}}^{R_{R_m}}} \Big(\Sigma^C(t_0 + T_{C_{Y_m}}^{I_{Y_m}}) - \Sigma^C(t_0 + T_{C_{Y_m}}^{I_{Y_m}} - T_{C_{Y_m}}^{R_{R_m}}) \Big) \right. \\ & \left. + \Sigma^C(t_0 + T_{C_{Y_m}}^{I_{Y_m}}) - \Sigma^C(t_0) \right], \end{aligned} \tag{22}$$

$$\begin{aligned} I_{Y_m}(t_0) = {} & \frac{N_{Y_m}(t_0)\, p_{I_{Y_m}}}{\sum_{m=0}^{k} N_{Y_m}(t_0)\, p_{I_{Y_m}}} \left[\mu_{I_{Y_m}}^{H_{R_m}} (\Sigma^C(t_0) - \Sigma^C(t_0 - T_{I_{Y_m}}^{H_{R_m}})) \right. \\ & \left. + (1-\mu_{I_{Y_m}}^{H_{R_m}}) \Big(\Sigma^C(t_0) - \Sigma^C(t_0 - T_{I_{Y_m}}^{R_{R_m}}) \Big) \right], \end{aligned} \tag{23}$$

$$\begin{aligned} H_{Y_m}(t_0) = {} & \frac{N_{Y_m}(t_0)\, p_{H_{Y_m}}}{\sum_{m=0}^{k} N_{Y_m}(t_0)\, p_{H_{Y_m}}} \left[\mu_{I_{Y_m}}^{H_{R_m}} \left(\mu_{H_{Y_m}}^{U_{R_m}} \Big(\Sigma^C(t_0 - T_{I_{Y_m}}^{H_{R_m}}) - \Sigma^C(t_0 - T_{I_{Y_m}}^{H_{R_m}} - T_{H_{Y_m}}^{U_{R_m}}) \Big) \right. \right. \\ & \left. \left. + (1-\mu_{H_{Y_m}}^{U_{R_m}}) \Big(\Sigma^C(t_0 - T_{I_{Y_m}}^{H_{R_m}}) - \Sigma^C(t_0 - T_{I_{Y_m}}^{H_{R_m}} - T_{H_{Y_m}}^{R_{Y_m}}) \Big) \right) \right], \end{aligned} \tag{24}$$

$$U_{Y_m}(t_0) = \frac{N_{Y_m}(t_0)\, p_{U_{Y_m}}}{\sum_{m=0}^{k} N_{Y_m}(t_0)\, p_{U_{Y_m}}} \sigma^U(t_0). \tag{25}$$

For the accumulated deaths per subpopulation, the full history of subpopulation sizes and deaths had to be known. Since D_{Y_m} or $N_{Y_m}^{D^\perp}$ are not consistently reported for the German counties and since the number of SARS-CoV-2 related deaths is small compared to the total population size, we only use one compartment and start with

$$D(t_0) = 0. \tag{26}$$

The extrapolated accumulated number of deaths for the total population until t_0 is approximated by

$$\widetilde{D}(t_0) = \Sigma^D(t_0 - T_{I_{Y_0}}^{H_{R_0}} - T_{H_{Y_0}}^{U_{R_0}} - T_{U_{Y_0}}^{D_{R_0}}), \tag{27}$$

since $T_{I_{Y_m}}^{H_{R_m}} = T_{I_{Y_n}}^{H_{R_n}}$, $T_{H_{Y_n}}^{U_{R_n}} = T_{H_{Y_n}}^{U_{R_n}}$, and $T_{U_{Y_m}}^{D_{R_m}} = T_{U_{Y_n}}^{D_{R_n}}$ for $0 \leq m, n \leq k+1$.

For the number of recovered and susceptible at t_0, we can now proceed as follows. Due to the simplifying assumption that multiple recovery from infections equates to recovery from a single infection and that recovery equates to full vaccination, we do not have separate compartments S_{Y_2}, R_{Y_0}, R_{Y_1}, or R_{Y_2} but only one immune compartment R; cf. Fig. 1 in [6]. This immune compartment is initialized by

$$R(t_0) = \min\left\{P,\, \max\left\{0,\, \Sigma^V(t_0-7) + \Sigma^C(t_0) - \sum_m \left(I_{Y_m}(t_0) + H_{Y_m}(t_0) + U_{Y_m}(t_0)\right) - D(t_0)\right\}\right\}. \tag{28}$$

Note that this more precise approximation of the recovered cases than $\Sigma^V(t_0-7)+\Sigma^C(t_0-14)$ (cf. (18)) can only be computed after (21)–(25) has been obtained with the help of N_{Y_m}, $m \in \{0,1,2\}$.

The number of partially vaccinated susceptibles (i.e., of $Y_1 = PV$) is set to

$$S_{Y_1}(t_0) = \max\left\{0,\, N_{Y_1} - E_{Y_1}(t_0) - C_{Y_1}(t_0) - I_{Y_1}(t_0) - H_{Y_1}(t_0) - U_{Y_1}(t_0)\right\}. \tag{29}$$

The number of unvaccinated susceptibles (i.e., of Y_0) at t_0 is set to

$$S_{Y_0}(t_0) = \max\left\{0,\, P - \sum_m \left(E_{Y_m}(t_0) + C_{Y_m}(t_0) + I_{Y_m}(t_0) + H_{Y_m}(t_0) + U_{Y_m}(t_0)\right) - S_{Y_1}(t_0) - R(t_0) - D(t_0)\right\}. \tag{30}$$

References

[1] Robert Koch-Institute. RKI Covid-19 Germany; 2021. Available from: `https://experience.arcgis.com/experience/478220a4c454480e823b17327b2bf1d4`.

[2] Robert Koch-Institute. COVID-19-Impfungen in Deutschland. Berlin: Zenodo. 2021;doi:10.5281/zenodo.5126652.

[3] Deutsche Interdisziplinäre Vereinigung für Intensiv- und Notfallmedizin (DIVI). DIVI Intensivregister; 2021. Available from: `https://www.intensivregister.de/#/aktuelle-lage/downloads`.

[4] Robert Koch-Institute. Nationale Teststrategie - wer wird in Deutschland auf das Vorliegen einer SARS-CoV-2 Infektion getestet? Robert Koch-Institute; 2021. Available from: `https://www.rki.de/DE/Content/InfAZ/N/Neuartiges_Coronavirus/Teststrategie/Nat-Teststrat.html?nn=13490888`.

[5] Kühn MJ, Abele D, Binder S, Rack K, Klitz M, Kleinert J, et al. Regional opening strategies with commuter testing and containment of new SARS-CoV-2 variants. medRxiv. 2021;doi:10.1101/2021.04.23.21255995.

[6] Koslow W, Kühn MJ, Binder S, Klitz M, Abele D, Basermann A, et al. Appropriate relaxation of non-pharmaceutical interventions minimizes the risk of a resurgence in SARS-CoV-2 infections in spite of the Delta variant. medRxiv. 2021;doi:10.1101/2021.07.09.21260257.
