# Supplementary material for: Appropriate relaxation of non-pharmaceutical interventions minimizes the risk of a resurgence in SARS-CoV-2 infections in spite of the Delta variant
Source: PLoS Comput Biol. 2022 May 16;18(5):e1010054. doi: 10.1371/journal.pcbi.1010054 (PMC9135349; doi:10.1371/journal.pcbi.1010054)
Supplement: Attachment — Submitted filename: ReviewerResponse2.pdf [file pcbi.1010054.s003.pdf]

Reviewer #2

PCOMPBIOL-D-21-01275: Appropriate relaxation of non-pharmaceutical interventions minimizes the risk of a resurgence in SARS-CoV-2 infections in spite of the Delta variant.

In this study the authors analyse the transmission dynamics of COVID-19 in Germany under NPI relaxation, an increasing number of vaccinated individuals and in the presence of Delta variant. After some simplifying assumptions, the authors conclude that there is still a great risk of another wave of infections if NPIs are lifted too early. The current version of the manuscript has major methodological issues that must be addressed as depicted below.

Answer: We thank the reviewer for his or her effort in providing comments on improving the paper. We have addressed all comments and highlighted changes made to the manuscript in blue. In order to account for the valuable and important remarks made by the reviewers, we have implemented a considerably extended model allowing for reinfections and infections after full vaccination. We have also changed the structure of the model introduction and extended the description where we formerly referred to other papers from our group to make the paper self-contained. Finally, we have set all statements in the context of pandemic development over the last months.

1. *It is clear from many reports that the efficacy of all vaccines are lower than 100% independent of the variant. Additionally, there are some evidences that vaccines induce more protection than naturally acquired infections (10.1126/scitranslmed.abi9915). The model design assumes 100% immunity for both naturally infected or vaccinated individuals. Although the authors state that they do not intend to provide precise forecasts, these assumptions might have a significant impact on model inferences and conclusions. Moreover, it seems to me that there are no methodological limitations which prevent the authors to account for more realistic scenarios where no full immunity is acquired after infection/vaccination. Please, clarify it.*
   Answer: Thank you for pointing out the potentially large impact of this simplification. We have now implemented a considerably extended model to account for possible infection after vaccination.

   We now no longer consider the vaccination effect to be 100% protective, but instead use observed effects for protection from infection, disease (i.e., symptomatic infection), and hospitalization. As no clear data are available with regards to any potential additional effects on protection from lethal outcomes, we assume that the protection from death is due to the cumulative effects of protection from infection, disease, and severe disease. Parameters are extracted from a recent living review (https://www.eurosurveillance.org/content/10.2807/1560-7917.ES.2021.26.41.2100920) as well as from [1] Sheikh A, McMenamin J, Taylor B, Robertson C, Public Health S, the EIIC. SARS-CoV-2 Delta VOC in Scotland: demographics, risk of hospital admission, and vaccine effectiveness. Lancet. 2021;397(10293):2461–2. [2] Bernal JL, Andrews N, Gower C, Gallagher E, Simmons R, Thelwall S, et al. Effectiveness of COVID-19 vaccines against the B.1.617.2 variant. [3] Stowe J, Andrews N, Gower C, Gallagher E, Utsi L, Simmons R. Effectiveness of COVID-19 vaccines against hospital admission with the Delta

(B. 1.617. 2) variant. Public Health England. 2021.

The text in our paper has been adapted accordingly.

2. *The authors assume that the vaccination capacity remains constant since the beginning of the simulations. This is a plausible assumption. On the other hand, although not stated in the text, the authors also implicitly assume that the number of individuals getting vaccinated remains constant over time. Like other countries, vaccination rates have substantially decreased in Germany after reaching 60% coverage and this fact might also impact model inferences. Please, clarify it and acknowledge limitations if needed.*
Answer: Although it is true that we cannot expect vaccination ratios of, e.g., 80 % in the near future in Germany (and thus will not simulate it), it is also true that vaccination ratios of 80 % or even 87 % have been reached in European countries such as Spain and Portugal.

   In accordance with the revision of our model, we have now also revised the vaccination process which is now based on the finely resolved data of `https://github.com/robert-koch-institut/COVID-19-Impfungen_in_Deutschland`. We now have provided detailed description on how to obtain local, age-resolved vaccination rates. For near-future scenarios, we assume the constant but slow increase in vaccinations as of the last weeks. Using this rather constant vaccinations numbers for the near future will not exceed vaccination coverages that are (for the moment) unrealistic for Germany.

3. *In equations 1 and 9, the term (Cj + Cpv,j) is multiplied by 0.5. This value is not described in the text and, apparently, it is missing in equation 2 and 10. Please, clarify it.*
Answer: Thank you for attentive reading and pointing out this typo. We have corrected this error and replaced the different factors by the variable $\widetilde{\beta}_{C,j}$ as it was already done in `https://www.medrxiv.org/content/10.1101/2021.04.23.21255995v1`.

4. *Equation 1 as presented does not account for (i) individuals getting vaccination, that is, all susceptible individuals strictly move from S (or Spv) to E (Epv) compartments and, (ii) C+ and I+ (which also contribute to the transmission levels). The same argument is applied to equation 9. Please, clarify it.*
Answer: The interaction between the susceptible and (partially) vaccinated population happens on a daily basis which is separate from the interactions of the differential equations. This process is described in equations in detail in the section on the vaccination process. The same applies for Compartments C+ and I+, whose inflow from C and I respectively is also computed separately from the differential equations. To ease the reading of our paper, we have extended the paragraph on commuter testing and for the sake of completeness, we have also rephrased the introduction of our spatially resolved model from `https://www.sciencedirect.com/science/article/pii/S0025556421000845` and `https://www.medrxiv.org/content/10.1101/2021.04.23.21255995v1`.

5. *It is not clear how the authors considered commuters. Equations 4 and 12 do not account for any fixed influx rate of those individuals. The current formulation as presented by the system of equations does not have any effect in the transmission dynamics if initial conditions for C+ and Cpv+ are zero. Please clarify how commuters were considered in the model dynamics and report the initial conditions for all model variables.*

   Answer: The commuter exchange happens on a daily basis and not in the differential equations. We have added the section on the spatially resolved model and a paragraph on commuter testing that essentially presents the description of `https://www.sciencedirect.com/science/article/pii/S0025556421000845` and `https://www.medrxiv.org/content/10.1101/2021.04.23.21255995v1` in condensed form.

6. *Equation 17 (dRi/dt) does not account for individuals receiving the second dose of the vaccine. Please, clarify it.*

   Answer: The vaccination process happens on a daily basis, as does the commuter exchange. This is not explicitly shown in the differential equations. Please also see the previous answers. We have now implemented a considerably extended model that allows for infection after full vaccination.

7. *Please, label and describe each panel of all multi-panel figures for better presentation and clarity. Figure 6 should be reformulated – the current form is difficult to understand. For better comparison between different scenarios, Figures 3, 5, 7 and 8 should have only one scale for Infected panels, as well as for ICU and Death panels. The scale for Figure 4 panels should also be standardized. The authors are reporting 50%CI instead of the commonly used 95%CI. If there is no strong justification for doing so, please report the 95%CI.*

   Answer: Please note that age-dependency and spatial resolution are important properties for a model to provide quantification of expected infection numbers for the near future. However, the more we move to simulation frames of 30, 60, or even 90 days, these models can still only provide qualitative insight since changing policies or changing behavior cannot be anticipated for 30, 60, or even 90 days and since uncertainty from the parameter estimations accumulates with each day. Nevertheless, we think that also these qualitative results are of interest.

   Please note that we do not pretend to be able to provide **valuable, i.e., narrow** 95 %-CI for these large time spans and that, on the other hand, we even consider this to be impossible. 95 %-CI for time spans of, e.g., 90 days would just give values somewhere between "nothing" and "everything". Providing the computed median results as well as the 25 % or 75 % percentiles can provide valuable qualitative insight into possible scenarios exceeding the near future of, e.g., two weeks. Including the naturally large 95 % CI only reduces the insight into the qualitative results of the median-50 % runs after 60 or 90 days.

8. *Why are the authors just presenting the age stratified results for the worst-case scenario (Fig 4)? Even considering the 50%CI, the model inferences go from around zero (difficult to*

visualize the minimum values) to a considerable increase in the number of cases for all ages. I understand the authors' caution, however, considering that the conclusions must be strictly based on the study's findings, this result does not support statements such as "we can safely assume that the number of infections will grow even faster after the end of the simulation by the end of August" or "if we go back to our pre-pandemic methods soon, these numbers are rapidly increasing".

Answer: Please note that we also provide age-stratified results for all scenarios in Figure 6. We did not see Figure 4 as something as important as to show it for all scenarios. We only wanted to provide some more insights for at least one scenario.

9. *The authors report that they obtained p* values from reference 11 (last sentence in page 5). I was not able to find values for pU and pD in the cited reference. Please, clarify it.*

   Answer: We have extended the previous model by possible reinfections or infections after vaccination. Then, also the probabilities that we use are based on four new scientific articles. For details, please see also answer to question 1.

10. *Equation 30 assumes that the Delta variant is 40% more infectious than the Alpha one, the text says 20-60% and the simulations considered 40 and 60%. Please, standardize and make it clear in the Methods section.*

    Answer: Thank you for pointing out this inconsistency. The "20%" were indeed a typo and copy and paste error. We consider Delta to be either 40 or 60 % more infectious. We have also added three new references to validate these choices.

11. *What do authors mean by "the number of individuals at the start of the simulations are extrapolated from the RKI-database and DVI-database" (Results – first paragraph)? Please, be specific about how and which data were obtained and from each database. This information should be added to the Methods section.* **Answer:** Please note that RKI and DIVI only report case numbers of positive tested individuals and individuals admitted to ICU. In order to obtain corresponding input data for, e.g., exposed or hospitalized individuals, this data has to be extrapolated. We compute, e.g., hospitalized individuals based on the probability to be admitted to ICU after hospitalization and by shifting time series of ICU admissions. This extrapolation is intuitive and a full description of the rather intuitive formulas would just extend the paper by two pages. We think that it is sufficient that interested readers have full access to these computations by looking at the code itself: `https://github.com/DLR-SC/memilio/blob/74-Expand-Model-for-Vaccination-Paper/cpp/epidemiology_io/secir_parameters_io.cpp`.

    We have added a corresponding reference to the paper.

12. *The authors should specify the incidence unit in the text. For example, in the passage "incidences of about 600", do authors mean 600 cases/100.000 people? People, always specify it on the text.*

Answer: We thank the reviewer for noting this inaccuracy in the description of our results. We have now added the specification such as "weekly infections per 100 000 individuals" on multiple occassions. However, in order to not bother the text flow to much, we do not repeat this for every single figure.

13. *What does exactly "correspond to Figure 2" as mentioned in the second line of page 12? Please, be clear about it.*
    Answer: Thank you for pointing this out. We now write "The first decrease can be explained through Fig. 2.

*It's recommended that the authors provide the code used in this model.

Reviewer #2: No Data and code are not available. → **Answer:** This important item has been addressed. The code is now fully accessible on GitHub at `https://github.com/DLR-SC/memilio`. Additionally, we provide data sets and data download tools to create the full set of input data to run our simulations.
