# Supplementary material for: Appropriate relaxation of non-pharmaceutical interventions minimizes the risk of a resurgence in SARS-CoV-2 infections in spite of the Delta variant
Source: PLoS Comput Biol. 2022 May 16;18(5):e1010054. doi: 10.1371/journal.pcbi.1010054 (PMC9135349; doi:10.1371/journal.pcbi.1010054)
Supplement: Attachment — Submitted filename: Koslow et al - Appropriate relaxation - revised - Review 2.pdf [file pcbi.1010054.s004.pdf]

Reviewer #2

PCOMPBIOL-D-21-01275: Appropriate relaxation of non-pharmaceutical interventions minimizes the risk of a resurgence in SARS-CoV-2 infections in spite of the Delta variant.

The current version of the manuscript still has methodological issues that must be addressed for greater clarity of the work.

Answer: We thank the reviewer for the effort of a detailed review of our revised paper. We have addressed all the points mentioned by the reviewer. In particular, we have added a multi-page appendix explaining the initialization of our model. We have completely reworked the vaccination distribution approach to consequently use less assumptions as proposed by the reviewer. In the same step, we have updated the age-resolved county population estimates coming from official sources. Furthermore, we have addressed all typos and minor items mentioned. We provide detailed answers in the following. Changes in the manuscript are highlighted accordingly.

- *In the response to reviewers, with regards to the number of individuals at the start of the simulations being extrapolated from the RKI-database and DVI-database, the authors say: "This extrapolation is intuitive and a full description of the rather intuitive formulas would just extend the paper by two pages." It may not be the best practice to rely on intuition and a brief description (or perhaps a supplemental material) would bring more clarity to the work. I respect the position of the authors thought.*

  Answer: We have now added a multi-page appendix to the paper that should describe well the initialization of the models.

- *Also in the response to reviewers, the authors mentioned they changed the term "0.5" (factor that multiplied the term (Cj+Cpv,j) in Equations 1,2,9,10,17,18) to "beta_Cj". The current version of the manuscript presents "epsilon_Cj" and "epsilon_Ij". The authors introduced these new parameters but did not present them in the text. They are only presented in Tables 1 and 2. What do they represent? What is the motivation to choose these forms ("sigmoidal curve from 0.5 to 1 on incidence 10 to 20" and "sigmoidal curve from* $[0.0; 0.2]$ *to* $[0.4; 0.5]$ *on incidence 10 to 150")? How and from where were they derived?*

  Answer: Since the parameter β is often used in another context of ODE-SIR models, we decided to change the notation from $\widetilde{\beta}$ to ξ to avoid confusion in naming. We already had replaced the occurrences of $\widetilde{\beta}$ in the resubmitted versions (tables as well as the equations). We however apologize that $\widetilde{\beta}$ was still mentioned in the response to the reviewers. The sigmoidal curves are not new with this paper. These represent incidence-dependent detection ratios for carrier and infected individuals. Ranges between 0.05 and 0.5 were already used in [1], assumed and fitted from Italian data. Further fits on German data had been conducted in [2] and [3].

- *Regarding the 95%CI, the authors' response to reviewers is reasonable w.r.t the argumentation about the fact that "the 25 % or 75 % percentiles can provide valuable qualitative insight into*

possible scenarios exceeding the near future...". *Building on that, the authors should not consider, although clearly stated and justified, only the worst case for the simulated scenarios. For example, considering scenario 1, it is stated (line 365) that "early opening leads to a rise of infections from about 6-16 July onward." This is the worst case shown in Fig 4. Even considering the 50%CI, we can infer from the model output that the cases can substantially rise or fall to something close to zero (hard to see), independently if the Delta variant is considered 40 or 60% more infectious than Alpha. The same argument for scenarios 2 and 3. In addition, graph scales chosen for Figs. 4, 5, 6 and 7 do not allow comparing model's predictions and the actual values during the first half of the simulation.*

Answer: We agree with the reviewer that we should not only consider the worst case. It is difficult to show all important aspects in a reasonable number of images and thus we decided to insert additional information that may be difficult to be seen from the already large number of images to the text. The sentence "early opening leads to a rise of infections from about 6-16 July onward." describes exactly what may be difficult to see. The median results indicate (although still decent) rise of infections from the second to third week of July onward. For a better understanding, we now write:

"The median run indicates that the early opening leads to a rise of infections from about 6-16 July onward."

or

"The median results of Fig. 7 on the top shows a clear downward trend in case numbers as well as in ICU admissions."

or

"In the median, this also holds for Delta-60% (Fig. **??**, bottom) up to mid of August."

In

"Fig. **??** on the bottom shows that even in the worst case, predicted by the 75%-percentile with Delta-60 %, the number of infections at the end of the simulation are close to the number of the detected cases. A similar argument can be given for the ICU admissions."

the term "worst case" is used in another context. Here, p75 percentile is still small compared to the other scenarios and we can infer that 75 % of the runs even predicted lower numbers.

We also added:

Please note that the following evaluation is qualitatively and essentially based on the median results. As with a simulation of 90 days, uncertainties accumulate. Many other scenarios are possible as the p25 and p75 percentiles indicate. Note however, that we sample all contact reduction factors from intervals. As contact reduction is one of the most important factors in mitigation, the median may correspond best to the contact reduction in the center of the given intervals while p75 and p25 percentiles result from higher or lower sampled contact

reduction. As it is difficult to present all subtle details in a reasonably limited number of images, we also provide additional information on the median results in the text.

- *It is not clear to me why equations for vaccinated (17 and 18) and unvaccinated (1 and 2) individuals present the same parameter "pho_i", while equations for partially vaccinated individuals (9 and 10) present a different value "pho_PV" (Tables 1 and 2 just present "pho_i_(0)"). Additionally, the authors say that certain parameters from equations 1-8 were reused in Equations 9-26 "without introducing new variable names" (lines 170-171). What does it mean? Which parameters? What was the reason for that? It is highly recommended that the description of the methods be as clear as possible.*

  Answer: We thank the reviewer for attentive reading. This was indeed a typo, lacking the first index V, i.e., $\rho_{V,i}$. Since rho is the transmission probability, this $\rho_{V,i}$ represents the probability of the transition from susceptible to exposed for fully vaccinated individuals. The corresponding reduction was given in the first equation of (30). For the sake of clarity, we now additionally write:

$$\rho_Y = p_{E_Y}\rho.$$

  Lines 170-171, should become clear when looking into the equations or figure 1. However, we now also write:

  "That means, that we use the time spans $T_{E_i}^{C_i}, T_{C_i}^{I_i}, T_{I_i}^{H_i}, T_{H_i}^{U_i}, T_{U_i}^{R_i}, T_{U_i}^{D_i}$ for the path of partially and fully vaccinated individuals."

- *Line 97: the authors define Zi := U_i=1 nSi,Ei,Ci,... as the set of all compartments of age group i. This definition of "Zi" seems to be, however, the set of all compartments of ALL age groups. At the same sentence, it is not comprehensible and not stated what "j" stands for in N_j_D.*

  Answer: We thank the reviewer for attentive reading. This was indeed a typo that was introduced with the extended description. The $\cup$ had to be removed and j had to be i for the sum of all living individuals of age group i, only. We have corrected it accordingly.

- *An iterative method (page 12) was used to correct the differences between county of reported and home location of vaccination. Although the authors give a reference to the code, the method does not seem to be validated elsewhere. I wonder if it converges to a reasonable/correct solution and if its final results are independent of the order in which weights are corrected. Unfortunately, I cannot evaluate this point.*

  Answer: The iterative method has been established with the revised paper. The question of a "correct" solution is quite difficult to answer since there is noch such thing as "ground truth data" available. We have taken the reviewers comments into consideration and decided to use an approach that uses less assumptions. We now build upon labor market regions [4, 5]

which were derived such that interactions within the regions are strong and connections with other outside regions are few. We now write:

"However, it is difficult to make precise assumptions on the true number of vaccinations per county.

Since country- or federal state-wide averages may be averaging out some local effects, we decided to use intermediate-sized region averages. For this, we use a set of labor market regions [4, 5] which are built such that interactions within the regions are strong and connections with other outside regions are few. We have taken the 34 regions as provided by [5]. Due to an unrealistically high number of reported vaccinations (i.e., more vaccinations than inhabitants) in the labour market region of Ulm, we aggregated the regions of Ulm and Stuttgart. The remaining 33 intermediate-sized regions are depicted in Fig. **??** (left). Over these regions, we have computed age-dependent averages of full vaccination (in general, this means second-dose vaccination) for every day since the beginning of the vaccination process in Germany. The averaged vaccination ratios for age group 18-59 as of November 02, 2021 is depicted in Fig. **??** (right)."

- *Equation 33 is different than in the previous version. In this current form, for any t between 0 and T_V_PV, $D1 < 0$. Please, clarify it.*

  Answer: First of all, please note that the right hand side of equation is not a product but the evaluation of the function $\mathcal{D}_2(x)$ at $x = t + T_{PV}^V$. So there do not appear any negative values.

  Please note that the equation should not have changed. We only inverted left and right hand side and renamed t_{gap} to T_{PV}^V. Unfortunately, the minus sign that appeared in the first revised version should be a plus sign. We have corrected this with this revision.

- *The authors are referencing to Table 2 when, perhaps, it was supposed to refer to Table 1 (lines 115, 226, ...)*

  Answer: We thank the reviewer for noting that the label of Table 2 had been repeating the label of Table 1. This has been corrected.

- *The authors start page 14 arguing about the relative infectiousness of Delta w.r.t. Alpha and state: "60% is potentially closer to the true value". In my opinion, this sentence is not under the scope of this work.*

  Answer: This sentence was more understood as a summary of the the previous original articles. However, we agree that this is not the scope of the article and we thus removed it.

- *Line344: the number "7" is duplicated.*

  Answer: We thank the reviewer for attentive reading. This reference has been corrected.

- *Line 353: "??"*

  Answer: We thank the reviewer for attentive reading. The missing reference has been updated.

References

[1] Khailaie S, Mitra T, Bandyopadhyay A, Schips M, Mascheroni P, Vanella P, et al. Development of the reproduction number from coronavirus SARS-CoV-2 case data in Germany and implications for political measures. BMC medicine. 2021;19(1):1–16.

[2] Kühn MJ, Abele D, Mitra T, Koslow W, Abedi M, Rack K, et al. Assessment of effective mitigation and prediction of the spread of SARS-CoV-2 in Germany using demographic information and spatial resolution. Mathematical Biosciences. 2021; p. 108648. doi:https://doi.org/10.1016/j.mbs.2021.108648.

[3] Kühn MJ, Abele D, Binder S, Rack K, Klitz M, Kleinert J, et al. Regional opening strategies with commuter testing and containment of new SARS-CoV-2 variants. medRxiv. 2021;doi:10.1101/2021.04.23.21255995.

[4] Kropp P, Schwengler B. Three-step method for delineating functional labour market regions. Regional Studies. 2016;50(3):429–445.

[5] Zika G, Schneemann C, Hummel M, Maier T, Kalinowski M, Bernardt F, et al. Langfristige Folgen von Demografie und Strukturwandel für regionale Arbeitsmärkte. IAB-Forschungsbericht; 2020.
